# Trichilemmal Keratinized Lesion of the Upper Palpebral Conjunctiva: A Case Report

**DOI:** 10.7759/cureus.88951

**Published:** 2025-07-29

**Authors:** Anastasia Papachristou, Andreas Papandroudis, Eleni Lagoudaki, Anna Detoraki, Efstathios T Detorakis

**Affiliations:** 1 Department of Ophthalmology, University Hospital of Heraklion, Heraklion, GRC; 2 Department of Nursing, University of Thrace, Didimoteicho, GRC; 3 Pathology Laboratory, Univeristy Hospital of Heraklion, Heraklion, GRC; 4 Department of Medicine, European University of Cyprus, Nicosia, CYP

**Keywords:** conjnuctiva, cyst, ocular surface, trichillemal lesion, tumor

## Abstract

Objective: To present a case of keratinized lesion of the upper palpebral conjunctiva in a patient without prior ocular or systemic history.

Methodology: A case of keratinized lesion located at the upper palpebral conjunctiva in a 21-year-old female patient was managed at the Department of Ophthalmology of the University Hospital of Heraklion. Comprehensive clinical assessments, surgical excision, histopathological examinations, and relevant investigations were conducted to characterize the lesion and identify clinical and histological features. The patient was followed up for 12 months postoperatively.

Results: Major presenting symptoms were ocular discomfort and conjunctival hyperemia. Histopathological examination was compatible with a cystic lesion with trichilemmal keratinization. No recurrence was observed during the follow-up period.

Conclusions: Focal keratinization of diverse pathogenetic origin and histological appearance at the epithelium of the upper palpebral conjunctiva can be found in young patients without a previous history of trauma, surgery, or inflammation.

## Introduction

Acquired conjunctival metaplastic lesions are usually associated with a history of previous trauma, surgery, or inflammatory reactions and may present with a variety of symptoms, including foreign body sensation, tearing, blurry vision due to secondary astigmatism, ocular pain or discomfort [1]. Such lesions are often cystic, resulting from obstruction of outflow in conjunctival glandular formations (retention cysts). The conjunctival epithelium is non-keratinizing, so a cyst containing keratin is considered atypical in the bulbar, fornical, or palpebral conjunctiva [2,3]. Moreover, keratin-containing cysts located in the palpebral conjunctiva are very rare because of the tight adherence of the thin substantia propria (subepithelial connective tissue layer) to the underlying tarsal plate. Until present, only two case reports on such cases have been published, with lesions affecting middle-aged male patients [2,3]. We present a case of a young female with keratin-containing cysts of the upper palpebral conjunctiva which displayed atypical histological features, such as trichilemmal keratinization (a distinctive pattern of keratin production resembling the outer root sheath of the hair follicle-specifically, the isthmic region), with to highlight the importance of clinical recognition of this rare condition.

## Case presentation

**Figure 1:**
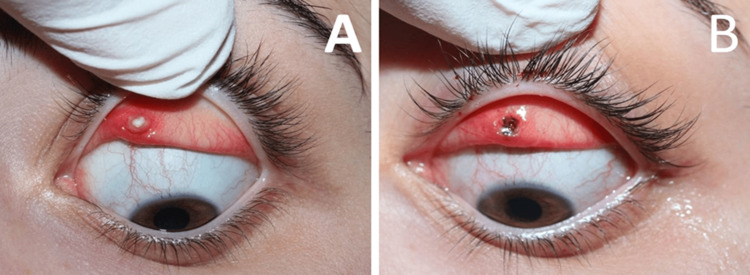
Preoperative (A) and immediate postoperative (B) appearance of the lesion site.

A 21-year-old female presented complaining of a foreign body sensation in her left eye for the previous two months, with progressive worsening. The patient reported using ocular surface lubricants for symptomatic relief. Previous ophthalmic and systemic history, including trauma, inflammatory reactions, or surgeries, as well as dermatological conditions, was unremarkable. Best-corrected visual acuity was 20/20 in both eyes (OU), and intraocular pressure was 12 mmHg in both eyes without medications. Biomicroscopy revealed vertical linear erosions in the upper cornea on the left eye, with mild fluorescein staining but without epithelial erosions or infiltrates. Mild conjunctival injection, mainly in the ipsilateral upper bulbar region, was also noted. Fundoscopy was unremarkable. Eversion of the left upper lid revealed a gray-white palpebral conjunctival mass, located at the medial third of the upper palpebral conjunctiva, with a size of 0.5 cm x 0.5 cm. The lesion was immobile and hard on palpation, causing localized conjunctival hyperemia (Figure 1A). The clinical appearance of the lesion was compatible with neoplastic or reactive mucosal conditions, such as papilloma or granuloma. The lesion was removed totally under topical anesthesia with cautery on the underlying tarsal bed of the upper eyelid (Figure 1B). Postoperatively, the patient received topical tobramycin-dexamethasone ointment three times a day (tid) for one week.

**Figure 2:**
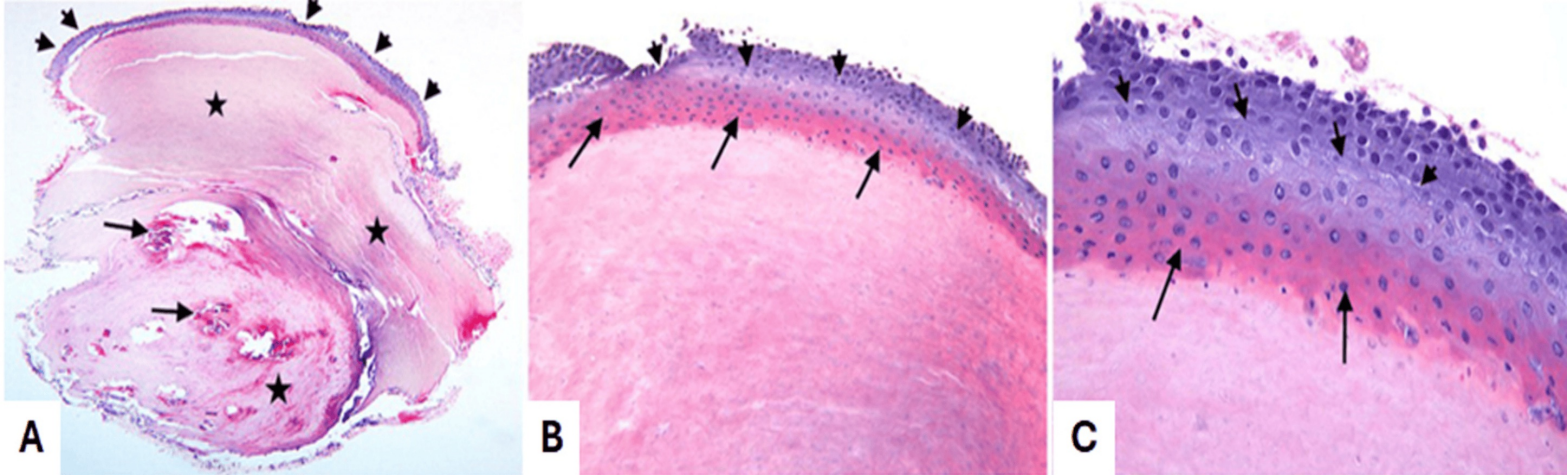
Histological appearance of the lesion. H/E stain magnification (A) x40, (B) x100, and (C) x200. Histopathologically, the cyst was lined by a multilayered squamous epithelium (arrowheads) and presented a cystic cavity containing a compact mass of amorphous keratin (star) with patchy foci of calcifications (arrows, A). The multilayered squamous epithelium comprised an outer layer of basaloid cells resembling the matrical cells in pilomatrixoma (arrowheads) and an inner layer of gradually maturing pale polygonal cells with eosinophilic cytoplasm displaying abrupt- trichilemmal-type keratinization (lacking keratohyalin granules) reminiscent the keratinization that takes place in the outer root sheath of hair (arrows, B, C).

The histopathological report described a keratin cyst with dimensions of 0.5 x 0.2 x 0.1. The amorphous mass of keratin was enclosed in a stratified squamous epithelium, which presented spontaneous keratinization and a spot of granular layer (stratum granulosum). No signs of cellular dysplasia or atypia were detected. The cyst was lined by a multilayered squamous epithelium (Figures 2A, 2C, arrowheads) and was structured as a cystic cavity containing a compact mass of amorphous keratin (Figure 2C, star) with patchy foci of calcifications (Figure 2C, arrows). The multilayered squamous epithelium comprised an outer layer of basaloid cells resembling the matrix cells in pilomatrixoma (Figures 2A-2C, arrowheads) and an inner layer of gradually maturing pale polygonal cells with eosinophilic cytoplasm displaying abrupt- trichilemmal type- keratinization (devoid keratohyalin granules) reminiscent the keratinization that takes place in the outer root sheath of hair (Figures 2B, 2C, arrows). No goblet cells were present. The aforementioned histopathologic characteristics were compatible with the diagnosis of trichilemmal keratin cyst. The excision was complete, and the residual conjunctival tissue had characteristics of chronic conjunctivitis. No signs of malignancy were detected, and the patient has been followed for 12 months postoperatively without recurrence.

## Discussion

The typical process of keratinization in the stratified squamous epithelium of the eyelid skin stops abruptly at the mucocutaneous junction. This junction lies just posterior to the excretory ducts of the meibomian glands. From this point, the eyelid epithelium retains its stratified squamous arrangement for approximately 1 mm. Beyond this point, it transitions into a stratified columnar arrangement. The number of cell layers gradually decreases to two, and mucous cells begin to appear [4]. The presence of keratin and keratinized epithelium above this point is considered abnormal, and processes such as inflammation, trauma, exposure, and drying of the conjunctiva, or neoplastic activity may cause ectopic keratinization. Jakobiec et al. described the existence of a keratinous cyst in the caruncle, resulting from an obstructed sebaceous gland duct. The cyst was lined by squamous epithelium elaborating trichilemmal-type keratin [5]. Congenital conjunctival dermoids may contain keratin and can be found in the subconjunctival space of the fornices [6]. In addition, intratarsal keratinous cysts are described in the bibliography in patients with Gorlin syndrome [7] or deriving from meibomian gland ducts [8]. 

In the case presented in this report, no previous ophthalmic or systemic history (such as ocular disease, trauma, or inflammation) was mentioned, in contrast to previous reports. Moreover, the patient was young and was under no systemic medications. To our knowledge, only two case reports of keratinous cysts of the palpebral conjunctiva are reported in the medical literature, both with complaints of ocular surface irritation, as in the case in this report [2,3]. Prominent corneal epithelial defects accompanied by epi-bulbar injection were described. The patients were first treated with antibiotics and eye lubricants, and reported no improvement in symptoms. The de-epithelialized exposed keratin acts as a hard irritant of the cornea and causes abrasions, which stain with fluorescein. In both previously reported cases, as in our case, the tarsus was not deeply infiltrated, and as a result, the superficial removal of the lesion did not affect the underlying tarsal surface. However, unlike the previously published cases, in the case presented in this report, the transition to keratin was characterized as trichilemmal type. Histopathologic features characteristic of trichilemmal keratinization, a heretofore undescribed metaplasia of the conjunctival epithelium were patchy calcifications, trichilemmal cyst with trichilemmal or abrupt keratinization without keratohyalin granules, keratinization in the outer root sheath of hair (trichilemmal) and peripheral layers demonstrating a palisading arrangement with swollen and filled with pale cytoplasm cells close to the cyst cavity. The cyst cavity contained amorphous eosinophilic keratin with foci of calcifications within the keratin. In both previous case reports, as in our case, no signs of malignancy were detected, and no recurrences were reported following surgical excision. Findings imply that the palpebral conjunctiva may develop highly atypical keratinous lesions, which could cause symptoms and necessitate prompt recognition with upper eyelid eversion, followed by complete surgical removal and histopathological evaluation.

## Conclusions

Although rare, keratinized lesions of the palpebral conjunctiva may be encountered even in young individuals and have atypical histological features, such as trichilemmal keratinization, as in the case presented here.
